# Real-World Eligibility for Germline Multigene Panel Testing in Breast Cancer: An Evaluation of Current Testing Criteria

**DOI:** 10.3390/diagnostics16142244

**Published:** 2026-07-17

**Authors:** Roxana Pintican, Nicoleta Antone Zenovia, Vlad Alexandru Gata, Natalia Moise, Andrei Roman, Carmen Lisencu, Adrian Pavel Trifa, Ovidiu Balacescu, Madalina Filip, Maria Miclaus, Andreea Catana, Catalin Vlad, Patriciu Achimas-Cadariu

**Affiliations:** 1 Department of Radiology, “Iuliu Hatieganu” University of Medicine and Pharmacy, 400012 Cluj-Napoca, Romania; pintican.roxana@gmail.com (R.P.); natymoise@yahoo.com (N.M.); andrei.roman678@gmail.com (A.R.); catanaandreea@gmail.com (A.C.); catalinvlad@yahoo.it (C.V.); patrick.achimas@hotmail.com (P.A.-C.); 2 Breast Cancer Center, The Oncology Institute “Prof. Dr. Ion Chiricuta”, 400015 Cluj-Napoca, Romania; carmen_lisencu@yahoo.com (C.L.); trifa.adrian@gmail.com (A.P.T.); m.miclaus@outlook.com (M.M.); 3 Department of Radiology, The Oncology Institute “Prof. Dr. Ion Chiricuta”, 400015 Cluj-Napoca, Romania; 4 Department of Oncology, The Oncology Institute “Prof. Dr. Ion Chiricuta”, 400015 Cluj-Napoca, Romania; 5 Department of Surgery, The Oncology Institute “Prof. Dr. Ion Chiricuta”, 400015 Cluj-Napoca, Romania; 6 Center for Research and Innovation in Personalized Medicine of Respiratory Diseases, Discipline of Medical Genetics, “Victor Babes” University of Medicine and Pharmacy, 300041 Timisoara, Romania; 7 Center of Expertise on Rare Pulmonary Diseases, Clinical Hospital of Infectious Diseases and Pneumophysiology “Dr. Victor Babes”, 300310 Timisoara, Romania; 8 Department of Genetics Genomics and Experimental Pathology, The Oncology Institute “Prof. Dr. Ion Chiricuta”, 400015 Cluj-Napoca, Romania; obalacescu@yahoo.com; 9 Department of Genetic Explorations, The Oncology Institute “Prof. Dr. Ion Chiricuta”, 400015 Cluj-Napoca, Romania; 10 Department of Medical Genetics, “Iuliu Hatieganu” University of Medicine and Pharmacy, 400012 Cluj-Napoca, Romania

**Keywords:** breast cancer, multigene panel tests, genetic criteria, NCCN, BRCA

## Abstract

**Background**: Germline multigene testing is increasingly integrated into breast cancer care, but the ability of current eligibility frameworks to identify pathogenic variant carriers remains uncertain in real-world cohorts. **Methods**: This retrospective observational study included 556 patients with histologically confirmed breast cancer who underwent germline multigene testing at a single oncology center. Testing eligibility was retrospectively assessed according to NCCN criteria, ESMO-based recommendations, and the ASBrS universal-testing recommendations. Family history up to third-degree relatives and surgical data were evaluated. **Results**: Pathogenic variants were identified in 137 patients (24.6%), while variants of uncertain significance were reported in 310 patients (55.8%). NCCN criteria were fulfilled by 135 of 137 patients with pathogenic variants (98.5%), indicating high sensitivity but limited discriminatory capacity, as eligibility was also frequent among patients without pathogenic variants. ESMO-based criteria were met by 94 mutation-positive patients (68.6%) and were significantly associated with pathogenic variant status (*p* < 0.001; OR = 3.08, 95% CI: 2.05–4.65). Notably, 42.3% of pathogenic-variant-positive patients reported no family history of malignancy. Prophylactic surgery was documented in only 16.8% of patients with pathogenic variants. **Conclusions**: NCCN criteria captured nearly all mutation-positive patients; ESMO-based criteria were more selective. The findings support broader access to germline testing before surgery.

## 1. Introduction

Breast cancer remains the most frequently diagnosed malignancy worldwide and a leading cause of cancer-related mortality among women [1,2]. Germline genetic alterations play a central role in hereditary breast cancer, with high-penetrance genes such as BRCA1, BRCA2, PALB2, TP53, PTEN, and CDH1, as well as moderate-risk genes, including CHEK2 and ATM, contributing significantly to cancer susceptibility [2,3,4]. The integration of germline genetic testing into routine oncologic care has become increasingly important, given its implications for risk assessment, therapeutic decision-making, cascade testing, and preventive strategies [2,5]. In particular, the identification of pathogenic or likely pathogenic variants may influence eligibility for targeted therapies, surgical planning, and risk-reducing interventions [6].

The clinical management of patients with pathogenic germline variants varies according to the affected gene. Current recommendations support discussion of risk-reducing mastectomy and/or risk-reducing salpingo-oophorectomy in carriers of pathogenic variants in BRCA1, BRCA2, and PALB2, whereas evidence remains insufficient to support similar recommendations for all other breast cancer predisposition genes [7]. In addition, pathogenic variants in TP53 may influence radiotherapy decisions, while BRCA1/2 pathogenic variants have direct therapeutic relevance through eligibility for PARP inhibitors in selected high-risk or advanced breast cancer settings [8,9,10].

As the clinical utility of germline testing continues to expand, optimizing patient selection for testing remains an important aspect of hereditary breast cancer care. International guidelines, including NCCN and ESMO recommendations, provide structured criteria for identifying individuals at increased hereditary risk [5,11]. However, emerging evidence suggests that guideline-based criteria may fail to identify a proportion of mutation carriers, particularly in unselected or heterogeneous real-world populations [12,13,14]. Therefore, the present study primarily aimed to evaluate germline testing eligibility among patients with breast cancer according to NCCN criteria, ESMO-based recommendations, and the American Society of Breast Surgeons (ASBrS) universal-testing recommendation. Secondary objectives included assessing the presence of variants of uncertain significance and evaluating the family history.

## 2. Materials and Methods

### 2.1. Study Design

This study was an analytical, observational, cross-sectional, retrospective study conducted at the Prof. Dr. I. Chiricuță Oncology Institute in Cluj-Napoca over the period October 2023–December 2025. Prior to study initiation, approval was obtained from the institutional Ethics Committee (Ethic Committee Name: Prof Dr. Ion Chiricuta Oncology Institute, Cluj-Napoca, Romania, 131/2024 with addendum 2025).

### 2.2. Participants and Data Collection

The study population consisted of patients with histologically confirmed breast cancer who underwent multigene germline genetic testing. Family history data were obtained from information recorded during genetic counseling consultations and subsequently extracted from medical records. Germline multigene panel testing was performed using clinically validated next-generation sequencing panels comprising either 125 or 130 cancer-predisposition genes, according to the testing protocol available at the time of analysis. The specific panel version used for each patient was recorded, and both panels included the major high- and moderate-penetrance genes associated with hereditary breast cancer predisposition. Pathogenic and likely pathogenic variants were classified according to established ACMG/AMP variant interpretation criteria, while variants of uncertain significance were reported separately and were not considered clinically actionable. Consecutive patients were included whether testing had been performed because of the institutional practice pathway, internationally accepted guideline-based indications (NCCN, ESMO), or patient preference.

For the purposes of this study, eligibility for genetic testing was retrospectively classified according to three frameworks: National Comprehensive Cancer Network (NCCN) criteria, ESMO-based recommendations, and the American Society of Breast Surgeons (ASBrS) consensus recommendations [15,16,17] (Table 1).

The ASBrS statement, which recommends that genetic testing should be made available to all patients with a personal history of breast cancer, was used as the broadest comparative framework. All patients underwent blood-based germline testing following pre-test genetic counselling, and all received post-test genetic counseling after disclosure of results. Clinical, surgical, and genetic data were extracted from the medical records, including age at diagnosis, personal and familial history, multigene test results (pathogenic/likely pathogenic variant; variant of uncertain significance—VUS) and surgery type. The family history was noted for up to third-degree relatives, including the spectrum of cancer types reported among relatives.

Broadly, internationally accepted indications for germline testing in breast cancer, as reflected in NCCN and ESMO guidance, include young age at diagnosis, triple-negative disease, male breast cancer, bilateral or multiple primary breast cancers, suggestive personal or family history of breast/ovarian/pancreatic/prostate malignancies, selected ancestry-associated risk settings, and clinical scenarios in which germline status may influence systemic treatment or surgical decision-making [15,16,17,18].

### 2.3. Statistical Analysis

Chi-square tests or Fisher’s exact tests were performed for each contingency table, with statistical significance set at *p* < 0.05. An individual Chi-square test was conducted for each of the 12 cancer types, comparing their distribution between patients and first-, second-, and third-degree relatives. To account for multiple comparisons, the Bonferroni correction was applied.

## 3. Results

### 3.1. Study Population and Genetic Testing Outcomes

A total of 556 patients with histologically confirmed breast cancer were included in the analysis. Age at diagnosis ranged from 23 to 75 years, with a mean age of 57 years. Pathogenic variants were identified in 137 patients (24.6%), whereas 419 patients (75.4%) had no pathogenic variants detected. Variants of uncertain significance were reported in 310 patients (55.8%), and other variants were identified in 73 patients (13.1%). These categories were not mutually exclusive: 88 (15.8%) patients carried both a pathogenic variant and at least one VUS. Accordingly, 222 patients had a VUS without a pathogenic variant. No statistically significant difference was observed in the overall distribution of pathogenic variant status across the analyzed categories (*p* = 1.0) (Table 2).

### 3.2. Fulfilment of Genetic Testing Criteria

The distribution of genetic testing eligibility differed according to the guideline framework applied. Among patients with pathogenic variants, 135 of 137 patients (98.5%) fulfilled NCCN criteria. Although the association between NCCN eligibility and pathogenic variant status approached statistical significance, it did not reach the conventional threshold (sensitivity 98.5%, *p* = 0.098; OR = 3.74, 95% CI: 0.87–16.12). In contrast, fulfilment of ESMO criteria was significantly associated with pathogenic variant status: 94 patients with pathogenic variants (68.6%) met ESMO-based indications, compared with 173 patients without pathogenic variants (41.4%) (*p* < 0.001; OR = 3.08, 95% CI: 2.05–4.65). The combined fulfilment of both NCCN and ESMO criteria showed an even stronger association with pathogenic variant positivity (*p* = 0.003; OR = 10.88, 95% CI: 1.44–82.34). Six patients with pathogenic variants (4.4% of pathogenic-variant-positive patients; 1.1% of the total cohort) were found to carry two pathogenic mutations (Table 3).

### 3.3. Family History of Cancer

Family history did not significantly differ between patients with and without pathogenic variants when analyzed according to the presence of affected first-, second-, or third-degree relatives (*p* = 0.52). Similarly, no significant difference was observed when comparing the presence of one versus two cancers among relatives (*p* = 0.86). Among patients with pathogenic variants, cancer was reported in first-degree relatives in 79 cases, second-degree relatives in 69 cases, and third-degree relatives in 22 cases. Notably, 58 of the 137 patients with pathogenic variants (42.3%) reported no relatives diagnosed with cancer, indicating the absence of a known family history of malignancy in a substantial proportion of mutation-positive patients (Table 4 and Table 5). Detailed data regarding the spectrum of cancer types reported among first-, second-, and third-degree relatives, as well as the distribution of familial cancers among patients with VUS, are provided in the Supplementary Material (Tables S1–S6). These analyses were considered exploratory and are therefore presented descriptively, given the limited clinical actionability of VUS and the potential for incomplete or heterogeneous family history reporting.

### 3.4. Prophylactic Surgery Among Patients with Pathogenic Variants

Among the 137 patients with pathogenic variants, 23 patients (16.8%) underwent prophylactic surgery, whereas 40 patients (29.2%) did not. Data regarding prophylactic surgery were unavailable for 74 patients (54.0%).

## 4. Discussion

This study aimed to evaluate, in a real-world breast cancer cohort, how different germline genetic testing eligibility frameworks identify patients carrying pathogenic or likely pathogenic variants. The main finding was that NCCN criteria captured almost all patients with pathogenic variants, whereas ESMO-based criteria were more selective and were significantly associated with pathogenic variant positivity. In this cohort, 137 of 556 patients carried pathogenic variants, corresponding to a detection rate of 24.6%. This relatively high rate likely reflects the tested population and supports the clinical relevance of germline testing in breast cancer care.

The performance of the eligibility frameworks differed substantially. NCCN criteria identified 135 of 137 patients with pathogenic variants, suggesting very high sensitivity in this cohort. However, NCCN eligibility was also frequent among patients without pathogenic variants, indicating limited discriminatory capacity. In contrast, ESMO-based criteria were fulfilled by 68.6% of patients with pathogenic variants and were significantly associated with pathogenic variant status. These findings suggest that ESMO-based criteria may enrich for pathogenic variant detection but may also miss a proportion of pathogenic-variant-positive patients. The combined fulfilment of NCCN and ESMO criteria was associated with higher odds of carrying a pathogenic variant; however, the wide confidence interval indicates limited precision, and this result should be interpreted cautiously.

These observations are consistent with previous studies showing that guideline-based testing criteria may not identify all carriers of clinically actionable pathogenic variants [12,13,14,19,20,21]. Hereditary breast cancer remains underdiagnosed in some settings because testing criteria can miss patients with pathogenic or likely pathogenic variants [22]. In a large cohort evaluating genes with actionable NCCN management recommendations, approximately 40% of patients with pathogenic or likely pathogenic variants did not meet current NCCN testing criteria [23]. Together with our findings, these data support the need for careful evaluation of testing strategies in real-world populations and highlight the potential role of broader testing approaches, particularly when results may influence treatment, surgical planning, or cascade testing [22,24].

Unlike NCCN and ESMO criteria, the ASBrS recommendation advocates universal germline testing for all patients with breast cancer [17]. Therefore, while this approach maximizes sensitivity by identifying all pathogenic variant carriers, it does so at the expense of substantially increasing the number of patients undergoing testing. Consequently, traditional comparisons based on eligibility performance are inherently limited, and the principal consideration becomes the balance between diagnostic yield and resource utilization. Nevertheless, universal or broader testing strategies may be clinically relevant because they reduce the risk of missing carriers who do not fulfil conventional criteria. This is particularly important when germline results are available before definitive surgery, as mutation status may influence the choice between breast-conserving surgery, unilateral mastectomy, and simultaneous contralateral risk-reducing mastectomy [25,26]. Timely preoperative testing may therefore help selected patients avoid a second surgical procedure and allow for more individualized surgical planning [27]. However, the implementation of broader germline testing strategies may be associated with important financial, logistical, and workforce challenges, particularly in middle-income countries where access to genetic counseling and testing remains limited. Future studies should evaluate the cost-effectiveness and healthcare-system impact of expanding testing eligibility beyond current guideline recommendations.

Family history analysis showed no significant difference between patients with and without pathogenic variants when first-, second-, and third-degree relatives were considered. Notably, 42.3% of patients with pathogenic variants reported no known family history of malignancy. This finding suggests that family history, although clinically important, may be insufficient as a standalone criterion for selecting patients for germline testing. In real-world practice, incomplete family history, small family size, limited knowledge of relatives’ diagnoses, and underreporting may all reduce the sensitivity of family-history-based selection [28,29]. Variants of uncertain significance (VUSs) are reported relatively frequently in the literature, particularly as the use of multigene panel testing has expanded. However, their clinical interpretation and the clinical utility of VUS findings are limited, and their management remains debated, especially when they are encountered in genes with variable penetrance or in patients without a clearly concordant phenotype. More than half of the patients in our cohort had at least one VUS finding. Although VUS results should not guide clinical management according to current recommendations, they may generate uncertainty for both patients and clinicians and increase the demand for specialized genetic counseling. This challenge is expected to become increasingly relevant as multigene panel testing expands in routine clinical practice. In this context, VUS should be interpreted cautiously, ideally within a multidisciplinary framework, and should not be used as the sole basis for major clinical decisions [30,31].

The rate of prophylactic surgery among patients with pathogenic variants was relatively low, with 16.8% undergoing risk-reducing procedures, while data were unavailable for more than half of pathogenic-variant-positive patients. Current recommendations support discussion of risk-reducing strategies in carriers of high-penetrance pathogenic variants, particularly in genes such as BRCA1, BRCA2, and PALB2 [5,7,11]. Previous studies have reported higher uptake rates of risk-reducing surgery among mutation carriers, although rates vary according to age, gene, counseling, access to care, and patient preference [25,26]. In our cohort, the high proportion of missing data limits interpretation, but the findings emphasize the importance of integrating genetic counseling and surgical decision-making early in the treatment pathway. These considerations are especially important in Central and Eastern European and other middle-income healthcare systems, where access to germline testing may be constrained by reimbursement, genetic counseling availability, laboratory capacity, and turnaround times. In this setting, timely preoperative testing may be particularly valuable, as germline results can influence the initial surgical strategy and may help selected patients avoid a second operation, such as delayed contralateral risk-reducing mastectomy. Thus, broader and earlier access to genetic testing may improve both individualized surgical planning and healthcare resource utilization.

This study has several limitations. First, it was retrospective and conducted in a single oncology center, which may limit generalizability. Second, the cohort included patients who underwent germline testing and therefore may not fully represent all patients diagnosed with breast cancer during the study period. Third, eligibility criteria were applied retrospectively and may have been affected by incomplete documentation, particularly regarding family history. Fourth, data on prophylactic surgery were unavailable for a substantial proportion of patients, limiting conclusions regarding the clinical impact of genetic testing on surgical management. Fifth, some subgroup analyses, including gene-specific, age at diagnosis, and patients carrying two pathogenic variants, were limited by small sample size. Finally, there is a lack of data on risk-reducing salpingo-oophorectomy (RRSO) uptake and identification of possible barriers (menopausal status, fertility concerns, access to genetic counselling, referral pathways, and healthcare system constraints).

In conclusion, this real-world study suggests that NCCN criteria may be more effective for minimizing missing pathogenic germline variant carriers, whereas ESMO-based criteria appear to be more selective in identifying patients at higher hereditary risk. However, reliance on guideline-based criteria alone may still fail to identify some patients harboring pathogenic variants. These findings support the value of broader access to germline testing in breast cancer care, particularly when results can inform preoperative decision-making, risk-reducing strategies, and cascade testing.

## Figures and Tables

**Table 1:** NCCN, ESMO and ASBrS recommendations for eligibility for genetic testing.

Evaluated Aspect	NCCN Guidelines	ESMO Guidelines	American Society of Breast Surgeons (ASBrS)
General approach	Testing based on clinicopathological and family-history criteria	Testing based on hereditary risk assessment and clinical context	Recommends universal genetic testing for all breast cancer patients
Patient eligibility	Patients fulfilling specific testing criteria	Patients selected according to hereditary risk factors	All breast cancer patients, regardless of age or family history
Age at diagnosis	Frequently ≤ 50 years considered for eligibility	Young age at diagnosis strongly considered	No age restriction
Family history	Major selection criterion	Important component of risk evaluation	Not required
Triple-negative breast cancer (TNBC)	Typically eligible, especially <60 years	Strong indication for testing	Automatically included through universal testing
Bilateral or multiple primary cancers	Important eligibility criterion	Included in risk assessment	Not required
Ovarian/pancreatic/prostate cancer family history	Increases likelihood of eligibility	Included in hereditary-risk evaluation	Not required
Genes commonly evaluated	BRCA1/2 ± extended hereditary panels	BRCA1/2 ± additional susceptibility genes	Supports multigene panel testing whenever appropriate
Guideline philosophy	Risk-adapted selective testing	Clinically integrated hereditary-risk stratification	Maximizing mutation-carrier detection
Main limitation	May miss mutation carriers without obvious family history	Potential underdetection in unselected populations	Higher costs and increased rate of VUS findings
Main advantage	Higher specificity	Strong integration into European oncology practice	Higher sensitivity for germline mutation detection

**Table 2:** Distribution of patients according to genetic testing results.

Category	Number of Patients	Percentage (%)
Patients with pathogenic variants	137	24.6
Patients without pathogenic variants	419	75.4
Patients with variant of uncertain significance (VUS)	310	55.8
Total	556	100

**Table 3:** Fulfilment of NCCN, ESMO, and ASBrS criteria for germline genetic testing.

Criteria	Patients with Pathogenic Variants (%)	Patients Without Pathogenic Variants (%)
NCCN—meet	135 (98.5%)	397 (94.7%)
—do not meet	2 (1.5%)	22 (4.8%)
ESMO—meet	94 (68.6%)	173 (1.4%)
—do not meet	43 (31.4%)	244 (58.5%)
ASB—TOTAL	137	417

NCCN—National Comprehensive Cancer Network; ESMO—European Society of Medical Oncology; ASBrS—American Society of Breast Surgeons.

**Table 4:** Frequency of relatives with cancer.

	First-Degree	Second-Degree	Third-Degree
Pathogenic variants	79	69	22
No pathogenic variants	179	187	45

**Table 5:** Number of relatives with one or two cancers.

	Pathogenic Variants	No Pathogenic Variants
Grade 1—1 cancer	66	148
Grade 2—1 cancer	49	141
Grade 3—1 cancer	20	40
Grade 1—2 cancers	13	31
Grade 2—2 cancers	20	46
Grade 3—2 cancers	2	5

No statistically significant difference (all *p* > 0.05).

## Data Availability

The anonymized datasets analyzed during the current study are available from the corresponding author upon reasonable request.

## Supplementary Materials

The following supporting information can be downloaded at: https://www.mdpi.com/article/10.3390/diagnostics16142244/s1, Table S1: Types of cancer reported among relatives of tested patients; Table S2: Number of cancers reported among Relatives of patients carrying two pathogenic variants; Table S3: Number of patients with positive familial history and pathogenic variants and VUS carriers; Table S4: Number of cancers in patients with positive familial history and pathogenic variants and VUS carriers; Table S5: Number of cancers reported up to third degree relatives of patients with VUS; Table S6: Statistical significance according to cancer type.
