# Supplementary material for: Real-World Eligibility for Germline Multigene Panel Testing in Breast Cancer: An Evaluation of Current Testing Criteria
Source: Diagnostics (Basel). 2026 Jul 17;16(14):2244. doi: 10.3390/diagnostics16142244 (PMC13409409; doi:10.3390/diagnostics16142244)
Supplement: Supplementary file 1 [file diagnostics-16-02244-s001.zip › diagnostics-4339177-supplementary.pdf]

Supplementary Materials

***Table S1**. Types of Cancer Reported Among Relatives of Tested Patients*

	**Breast**	**Colorectal**	**Lung**	**Prostate**	**Melanoma**	**Ovarian**	Pancreas	Gastric	Hepatocellular	Uterine	Other
Grade 1	31	6	3	8	3	11	12	6	2	6	19
Grade 2	46	13	5	6	1	11	7	8	1	3	21
Grade 3	16	3	0	1	1	1	1	0	0	1	1

***Table S2**. Number of Cancers Reported Among Rela ;tives of Patients Carrying Two Pathogenic Variants*

	**Grade 1**		**Grade 2**		**Grade 3**	
	**1 cancer**	**2 cancers**	**1 cancer**	**2 cancers**	**1 cancer**	**2 cancers**
2 pathogenic variants	5	1	6	0	0	0
No pathogenic variants	148	31	141	46	40	5

No statistically significant differences were observed for Tables S1 and S2 (p>0.05).

Table S3. Number of patients with positive familial history and pathogenic variants and VUS carriers.

	**Grade 1**	**Grade 2**	**Grade 3**
Pathogenic + VUS	35	38	15
No pathogenic variants	179	187	45

Table S4. Number of cancers in patients with positive familial history and pathogenic variants and VUS carriers.

	**G**rade 1		**G**rade 2		**G**rade 3	
	1 cancer	2 cancers	1 cancer	2 cancers	1 cancer	2 cancers
Pathogenic + VUS	29	6	25	13	13	2
No pathogenic variants	148	31	141	46	40	5

No statistically significant differences were identified

Table S5. Number of Cancers Reported up to Third-Degree Relatives of Patients with VUS

	Grade 1		Grade 2		Grade 3	
	1 cancer	2 cancers	1 cancer	2 cancers	1 cancer	2 cancers
VUS	29	6	106	39	33	6
No pathogenic variants /VUS	57	9	57	18	19	1

Chi-square test of independence , with individual pairwise comparisons adjusted for multiple testing using Bonferroni correction. A highly significant association was observed ($p < 0.000001$), although most pairwise comparisons were not statistically significant.

Table S6. Statistical significance according to cancer type

**Cancer type**	**p-value**	**Significant after Bonferroni**
Breast	0.000000	Yes
Colorectal	0.000010	Yes
Lung	0.010053	No
Prostate	0.000449	Yes
Melanoma	0.118280	No
Ovarian	0.000003	Yes
Pancreatic	0.000010	Yes
Gastric	0.000267	Yes
Hepatocellular	0.217300	No
Uterine	0.006523	No
Other	0.000000	Yes

Chi-square test of independence with Bonferroni correction threshold ($\alpha = 0.0042$).
